# Epigenomics and Transcriptomics in the Prediction and Diagnosis of Childhood Asthma: Are We There Yet?

**DOI:** 10.3389/fped.2019.00115

**Published:** 2019-04-02

**Authors:** Erick Forno, Juan C. Celedón

**Affiliations:** ^1^Division of Pulmonary Medicine, UPMC Children's Hospital of Pittsburgh, Pittsburgh, PA, United States; ^2^Department of Pediatrics, University of Pittsburgh School of Medicine, Pittsburgh, PA, United States

**Keywords:** childhood asthma, epigenetics, transcriptomics, DNA methylation, RNA sequencing

## Abstract

Asthma is the most common non-communicable chronic disease of childhood. Despite its high prevalence, to date we lack methods that are both efficient and accurate in diagnosing asthma. Most traditional approaches have been based on garnering clinical evidence, such as risk factors and exposures. Given the high heritability of asthma, more recent approaches have looked at genetic polymorphisms as potential “risk factors.” However, genetic variants explain only a small proportion of asthma risk, and have been less than optimal at predicting risk for individual subjects. Epigenomic studies offer significant advantages over previous approaches. Epigenetic regulation is highly tissue-specific, and can induce both short- and long-term changes in gene expression. Such changes can start *in utero*, can vary throughout the life span, and in some instances can be passed on from one generation to another. Most importantly, the epigenome can be modified by environmental factors and exposures, and thus epigenetic and transcriptomic profiling may yield the most accurate risk estimates for a given patient by incorporating environmental (and treatment) effects throughout the lifespan. Here we will review the most recent advances in the use of epigenetic and transcriptomic analysis for the early diagnosis of asthma and atopy, as well as challenges and future directions in the field as it moves forward. We will particularly focus on DNA methylation, the most studied mechanism of epigenetic regulation.

## Epigenomics and Transcriptomics in the Prediction and Diagnosis of Childhood Asthma: Are We There Yet?

Asthma affects over 300 million individuals worldwide and it is the most common chronic disease of childhood (1). Total asthma costs exceed ~$81 billion per year in the United States (U.S.) (2) and ~72€ billion in Europe (3, 4). In the U.S. alone, asthma affects over 6.1 million children, and leads to 1.7 million emergency department (ED) visits, 136,000 hospitalizations, and over 10.5 million missed school days per year (5, 6). Asthma has a significant hereditary component, with a recent large meta-analysis of twin and sibling studies calculating its heritability (*h*^2^) at ~53% (7–10). Despite this, studies of genetic markers to date have only been able to explain a small proportion of disease risk—a phenomenon dubbed the “missing heritability” of complex diseases (11, 12). For example, a recent meta-analysis of genome-wide associations studies (GWAS), including 23,948 asthmatics and 118,538 controls, identified 18 novel loci associated with asthma, which overall explained only ~3.5% of variance in the risk of asthma (13). Predicting an individual's risk of asthma exacerbations or response to asthma medications using genetic markers has proven difficult as well (14–17). In general, analyses of single nucleotide polymorphisms (SNPs) have yielded small risk effect estimates and have failed to correctly identify risk (e.g., the probability of developing asthma, or the risk of exacerbations among affected subjects) at the individual level. More importantly, an individual's genetic code is stable and does not change in response to environmental factors or treatment interventions, and thus SNPs and other genetic markers are ill suited to predict an individual's varying risk of asthma-related outcomes over time.

Unlike genotype, epigenetic regulation and gene expression are responsive to environmental factors, and could thus bridge the gap between an individual's genetic predisposition and the environment to which they are exposed—whether this is *in utero*, in early life, or later on. This area of study has started to gain attention in asthma research and has shown significant promise to date. Here, we will first review the existing literature and then discuss challenges and potential future directions in the field.

## Brief Overview of Epigenetic Regulation

**Figure 1:**
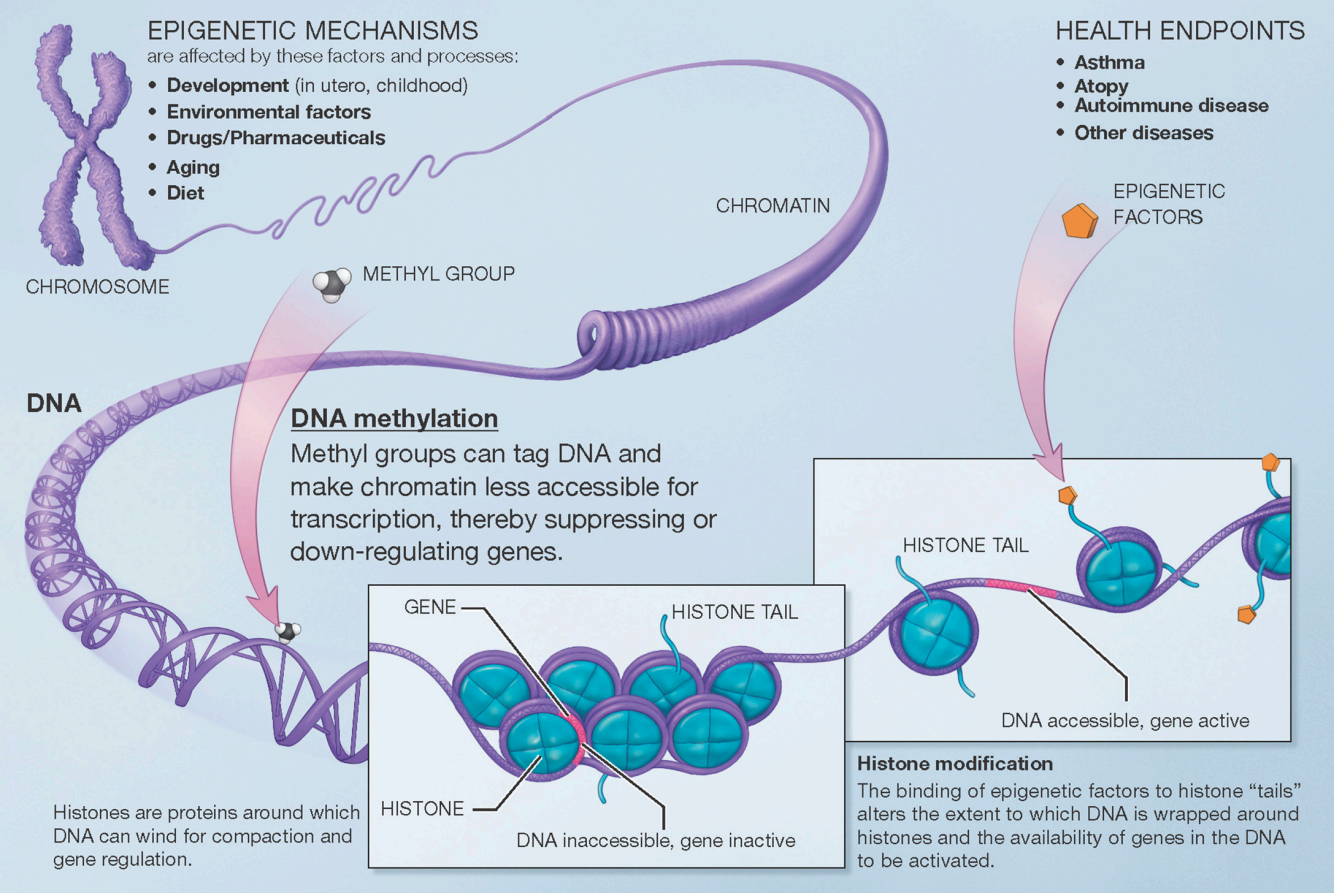
Overview of mechanisms involved in epigenetic regulation of gene expression. There are several epigenetic mechanisms that regulate gene expression during development and in response to environmental and other factors. DNA methylation suppresses (down-regulates) gene expression. Histone modification can occur via different pathways that lead to different effects (e.g., histone acetylation facilitates gene expression). A third group of mechanisms, post-translational RNA modification, is not pictured here for clarity. Modified from the U.S. National Institutes of Health (https://commonfund.nih.gov/epigenomics/figure).

Broadly speaking, the term “epigenetics” encompasses the regulation of cell activity and gene expression by mechanisms that do not alter the genetic code (DNA sequence) itself. The epigenome comprises all the chemical compounds and factors added to an individual's genome to regulate the expression of the genes within that genome. Epigenetic processes regulate the function of specific cells and tissues over time and in response to the environment, aging, and other factors, despite the fact that all cells in the human body contain the same genetic sequence. Here, we use the term “epigenomic” to encompass both epigenetic regulation and the resulting changes in gene expression. There are several known mechanisms of epigenetic regulation, of which DNA methylation and histone modifications are the best understood to date (Figure 1).

DNA methylation occurs throughout the genome at CpG sites, where a cytosine is immediately followed by a guanine in the 5′ to 3′ direction (the name “CpG,” which is short for a 5′-cytosine-phosphate-guanine-3′ dinucleotide, helps differentiate from “CG,” where a cytosine and guanine are on complementary strands of DNA). Methyl groups (CH_3_) are added to cytosine bases, resulting in 5-methylcytosine residues. These physically protrude into and obstruct the major groove of the DNA helix, while indirectly facilitating histone deacetylation. DNA methylation and histone deacetylation result in chromatin that is more compact and less accessible, generally leading to suppression or down-regulation of gene expression. Furthermore, while CpG sites exist throughout the genome, they occur with much higher frequency in areas denominated CpG islands, which are often located near the promoter regions of genes. Higher methylation in these islands also makes it more difficult for transcription factors to assemble, further down-regulating expression of the corresponding genes. The end-result of DNA methylation is thus often marked down-regulation of gene expression. Beyond this down-regulation of gene expression, however, DNA methylation outside of gene promoters may have functional consequences that are poorly understood; DNA methylation may also, although less frequently, occur at non-CpG sites. To date, most human studies of the epigenomics of asthma have focused on CpG DNA methylation and gene expression.

## Studies of DNA Methylation and Childhood Asthma

Some of the complex biological processes that contribute to the development of asthma may start *in utero*. Maternal smoking during pregnancy is a well-known and significant risk factor for childhood asthma. A recent meta-analysis of epigenome-wide association studies (EWAS), which included 6,685 children from 13 birth cohorts, reported that maternal smoking was associated with over 6,000 differentially methylated CpG sites in newborn blood at a genome-wide significant level (i.e., *P*-value corrected for multiple testing) (18, 19). Moreover, all such CpG sites showed at least nominal association (i.e., *P* < 0.05 without correction for multiple testing) at an average age of 6.8 years, indicating that methylation changes induced by maternal smoking during pregnancy can be long-lasting and extend into school age.

In a case-control study (nested in a larger birth cohort designed to study the maturation of the immune system in infancy), DeVries et al. looked at genome-wide DNA methylation in cord blood mononuclear cells (CBMCs) and asthma status by 9 years of age, and reported several hundred differentially methylated regions (DMRs); of those, the CpG markers in gene *SMAD3* remained significant even after adjusting for CBMC cell composition, and they replicated their findings in two independent cohorts (20). While the sample size was rather small (18 cases and 18 controls), this is one of very few longitudinal studies in children. Of interest, *SMAD3* DNA methylation has also been associated with atopic asthma (at age 5 years) in a study of children with a history of severe adenovirus-induced wheezing in infancy (21).

More recently, an EWAS of whole-blood DNA from four European cohorts (including 392 children with asthma and 1,156 children without asthma) identified 27 CpG sites that showed differential methylation at a genome-wide significant level (false discovery rate [FDR] *P* < 0.05); after using a looser threshold (FDR *P* < 0.10) for the preschool asthma outcome, the authors selected 35 CpGs for replication, of which 27 were available in the other cohorts. Of those 27 candidate CpG sites, 14 replicated in an independent sample (including 3,196 children with and without asthma), with joint *P* ranging from 3.7 × 10^−9^ to 2.6 × 10^−17^ (22). Moreover, the authors reported that methylation was lower (and associations stronger) in eosinophils than in other cell types, indicating that signals in whole blood may have been at least partially driven by eosinophil count. The authors also evaluated the 14 replicated CpG sites in cord blood in a subset of 1,316 children, but none of them was significantly associated with the development of asthma by age 3-4 years—suggesting that these methylation changes occur after birth, either in response to environmental factors that increase asthma risk, or perhaps following the development of asthma.

In another recently published meta-analysis of EWAS, the Pregnancy and Childhood Epigenetics (PACE) consortium examined the relation between blood DNA methylation at the newborn stage and asthma development by school age in eight European birth cohorts (23). The meta-analysis included cohort-level estimates for 3,572 children (668 with asthma and 2,904 controls) and identified nine significant CpG sites (FDR *P* < 0.05) and 35 DMRs. Of interest, the study also evaluated whole-blood DNA methylation later in childhood and found 179 CpG sites significantly associated with asthma (FDR *P* < 0.05), none of which overlapped with the nine markers that were significant in the newborn blood analysis. None of the nine newborn CpG sites was even nominally significant in the childhood analysis and, conversely, only two of 179 childhood CpG sites were nominally significant and in the same direction of association as in the newborn analysis. This further emphasizes that methylation markers for asthma risk may vary over time. Pathway analysis of the newborn CpG sites and DMRs showed enrichment for canonical pathways associated with immune function in asthma, such as the inflammasome and NF-kB signaling.

Similarly, Arathimos et al. evaluated DNA methylation and asthma in a subsample of ~800 children from the ALSPAC cohort at two time points: 7.5 and 16.5 years of age (*n* = 149 and *n* = 184 with asthma at each respective age) (24). They found the largest number of significant associations at 7.5 years of age (*n* = 411 differentially methylated CpGs at FDR *P* < 0.05), but none remained significant after adjusting for imputed differential white blood cell counts. At 16.5 years of age, CpGs in genes *AP2A2* and *IL5RA* were associated with asthma both before and after adjustment for cell counts. The authors also performed a bidirectional longitudinal analysis (asthma at 7.5 years vs. methylation at 16.5 years; and methylation at 7.5 years vs. asthma by 16.5 years), but neither showed significant CpGs after adjustment for cell counts. This highlights the importance of considering the tissue- and cell-specificity of epigenetic regulation.

Epigenomic studies have also helped better understand the role of genes identified through genetic studies such as GWAS. The *GSDMB/ORMDL3* locus on chromosome 17q21 is the most consistently replicated genetic region associated with asthma (25, 26). We now know that CpG SNPs (i.e., SNPs that exist as part of a CpG site in which methylation changes can occur) in that locus are associated with asthma, and that DNA methylation in the *ORMDL3* gene is associated with asthma, but that both SNPs and CpG sites are independently associated with *ORMDL3* gene expression (27). In other words, the association between the SNPs in the gene and the expression of the gene itself is likely not mediated through changes in DNA methylation. Future studies will be needed to further dissect the genotype-DNA methylation-gene expression relationships in this complex asthma susceptibility locus.

## Studies of DNA Methylation in Nasal Epithelium and Asthma

Blood plays a central role in immune regulation and other systemic processes, but studying the epigenomics of the airways will likely yield very important and complementary knowledge. Clustering analysis of DNA methylation has shown that the “distance” (a measure of dissimilarity) between the bronchial epithelium and blood samples is *twice* the distance between bronchial and nasal epithelia (28). This makes biological sense, as one would expect bronchial and nasal airway epithelial cells to be much more closely related to each other than to any of the cell types found in blood. Indeed, DNA methylation and gene expression in nasal epithelium are highly correlated with those in bronchial epithelium, particularly in non-smokers (28–31). In children with asthma, DNA methylation of candidate genes (such as *IL6* [IL-6] and *NOS2* [inducible nitric oxide synthase]) in nasal epithelium is associated with exhaled nitric oxide (FeNO), a marker of airway eosinophilic inflammation (30). Likewise, nasal epithelial samples from patients with cystic fibrosis (CF) show differential methylation of genes known to be associated with the severity of CF lung disease (31). Nasal epithelium is easily accessible by non-invasive means and has therefore become a very promising surrogate to study the epigenomics and transcriptomics of the lower airways.

In the first EWAS looking at DNA methylation in nasal epithelium and asthma, Yang et al. compared methylation profiles between 36 African-American children with atopic asthma and 36 non-atopic non-asthmatic control subjects (32). In that study, there were 186 differentially methylated genes, including several related to asthma, atopy, and immune responses. Of interest, none of the CpG sites or DMRs identified in nasal epithelium were significant in a similar analysis of peripheral blood mononuclear cells (PBMCs), highlighting both the cell specificity of epigenetic mechanisms and the advantages of studying nasal epithelium, which is more exposed to environmental factors that would affect and trigger asthma-related processes (i.e., allergens or viral infections).

**Figure 2:**
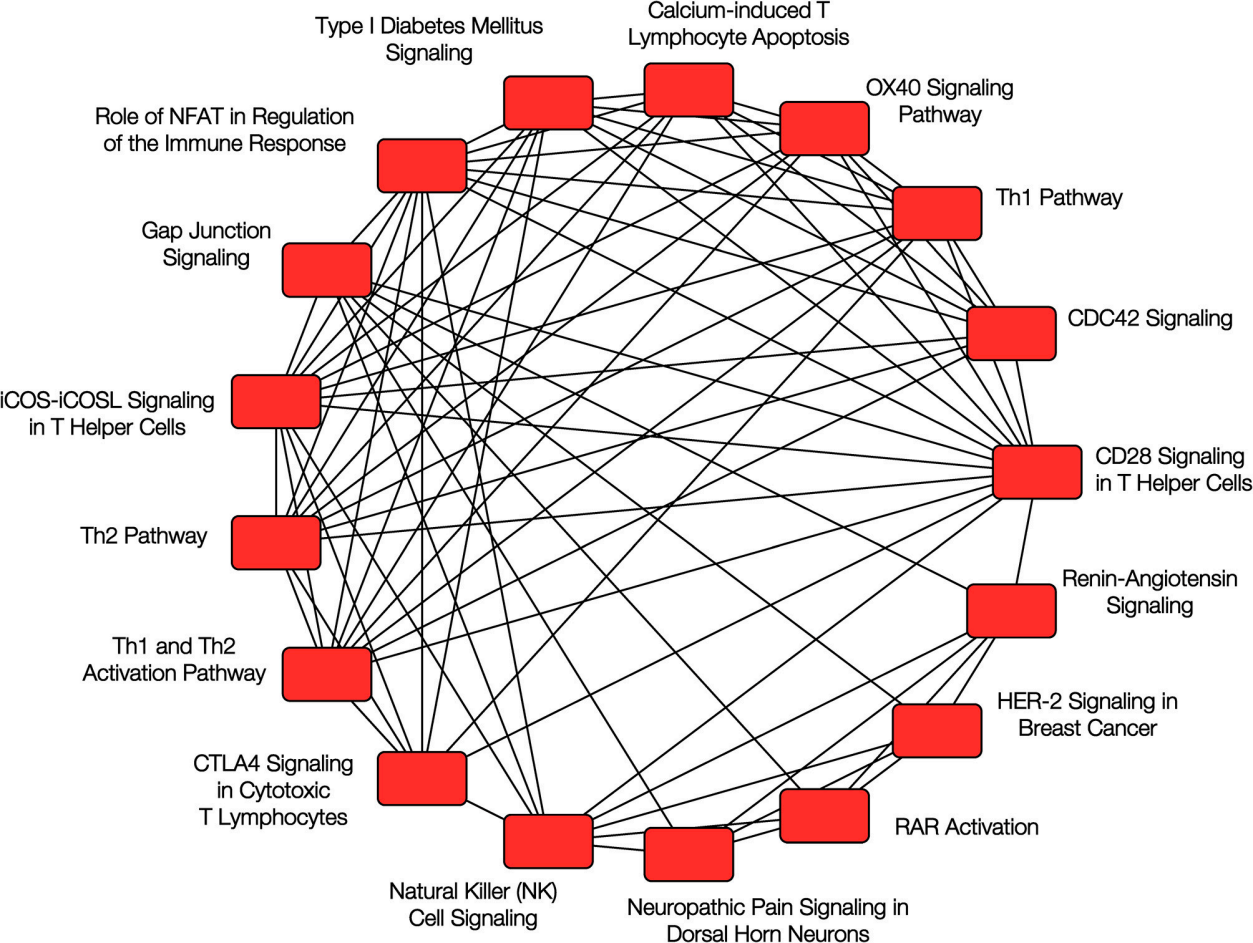
Pathway analysis of nasal epithelium epigenomics and atopy. Overlapping canonical pathways enriched in the analysis of DNA methylation and gene expression in nasal epithelium and childhood atopy. Reproduced with permission from Forno et al. (33), ©The Lancet Respiratory Medicine 2018 (https://doi.org/10.1016/S2213-2600(18)30466-1).

More recently, we evaluated the nasal DNA methylome in a cross-sectional study of 483 Puerto Rican children and identified over 8,600 CpG sites that showed differential methylation between children with and without atopy (33). Among the top 30 CpG probes (with FDR *P*-values between 9.6 × 10^−17^ and 2.2 × 10^−22^), 29 replicated in the same direction using the data in African-American children from Yang et al. and 28 replicated using data from 432 children from PIAMA, an unselected cohort of Dutch children; the joint *P*-values for the three cohorts ranged from 1.7 × 10^−19^ to 1.1 × 10^−47^. Many of the top genes we reported were related to epithelial barrier integrity or function and to immune regulatory processes (see Figure 2), including *CDHR3* (cadherin receptor 3, a receptor for rhinovirus C that has been implicated in asthma), *CDH26* (cadherin 26, a protein involved in CD4+ T cell regulation and in airway epithelial cell structure and polarity) (34), *GJA4* (gap junction protein alpha 4), *CAPN14* (a susceptibility locus for eosinophilic esophagitis that is induced by IL-13 and is also involved in epithelial repair) (35), *MTRNL* (which is over-expressed in atopic dermatitis) (36), *SLC9A3* (which has been linked to decreased lung function in CF) (37, 38), *FBXL7, PCSK6*, and others. We found considerable overlap between the CpG sites associated with atopy and those associated with atopic asthma.

In that study, we also showed substantial concordance between DNA methylation and gene expression, including 779 hyper-methylated CpG sites corresponding to 514 down-regulated genes and 1,506 hypo-methylated CpG probes corresponding to 815 up-regulated genes. Moreover, we built a 30-CpG panel that accurately classified 85% of Puerto Rican children according to their atopy status [with area under the curve [AUC] = 0.93–0.94], and 88% when comparing atopic asthma vs. non-atopic non-asthmatic controls (AUC = 0.95–1.00); the classification panel also performed remarkably well in both independent replication cohorts (accuracy 73 to 86% and AUC = 0.73–0.92) (33). In addition, we were able to replicate the top findings by Yang et al. (32) and the top nasal epithelium findings from Xu et al. (22). This study illustrates the marked potential of studying the nasal respiratory epithelium, as well as the robustness of results across populations from different racial/ethnic backgrounds that are exposed to diverse environments. If replicated in longitudinal studies, the nasal methylome and transcriptome could represent an important biomarker to identify infants and children at high risk of developing asthma or other asthma-related outcomes.

Some studies have also tried to use epigenomics to evaluate or predict response to asthma medications. In a pilot study of 33 children presenting to the ER with acute asthma exacerbations, four CpG sites near gene *OTX2* were differentially methylated (before and after steroid administration) between children who did and did not improve (time^*^treatment interaction) (39). In another small study, nasal epithelial cells from children with controlled, uncontrolled, and no asthma were cultured in the presence of dexamethasone, and the authors reported that upregulation of the gene *FKBP51* was blunted in samples from children with uncontrolled asthma (40). Although it is unclear whether this was due to the baseline therapy these children were receiving (on average, 1,000 μg of fluticasone/day), the study underscores the potential applications of epigenomics in evaluating or predicting medication response.

## DNA Methylation in Buccal, Saliva, or Sputum Samples

An early study evaluated DNA methylation in buccal cell samples from 37 pairs of monozygotic twins discordant for asthma at age 10, as well as the 13 pairs who remained discordant by 18 years of age (41). The authors reported several nominal associations, but none was significant after Bonferroni correction for multiple testing. The lack of significant results may be partly explained by the use of buccal cell samples (which, unlike immune or airway epithelial cells, may not be involved in asthma pathogenesis); and to limited statistical power—particularly if one considers that epigenetic regulation “predisposition” may be at least partly encoded in an individual's genetic code (which is shared by monozygotic twins). To our knowledge, only two studies have evaluated genome-wide DNA methylation in saliva samples. One studied 26 children with “respiratory allergy” (defined as a positive IgE to allergens plus report of asthma, wheeze, hay fever, rhinitis, or a runny nose) and 20 controls; after technical confirmation and replication in a separate sample, the authors reported that a DMR in the promoter for gene *GLI2* was consistently associated with the phenotype (42). The second study evaluated the association between DNA methylation in saliva at age 6–18 months and wheezing by age 18 months (68 cases and 68 controls); the authors reported a significant DMR containing 10 CpGs in gene *PM20D1* (43). We are not aware of any studies of childhood asthma using sputum samples.

## Studies of Gene Expression and Asthma

Gene expression studies in blood and nasal epithelial samples have also shown some promising results. In 2012, a small study in 27 atopic children and 11 controls reported 24 differentially expressed genes with a log2 (ratio) > 1, including several genes with biological relevance to atopy, such as *POSTN* (periostin) and *CST1* (cystatin SN) (44). While profiling based on the differentially expressed genes did not distinguish children with allergic rhinitis from those with asthma, it did show 75% accuracy in discriminating children with controlled and uncontrolled asthma. More recently, a study in 190 adults (66 with asthma and 124 without asthma) identified a profile of 90 differentially expressed genes in nasal epithelium that classified subjects based on their asthma status with high accuracy (AUC = 0.99) (45). Furthermore, the gene panel showed good discrimination against other (i.e., non-asthma) respiratory conditions such as upper respiratory infections (URIs), allergic rhinitis, cystic fibrosis, and smoking.

In terms of specific and previously known risk factors for asthma, several studies have looked at transcriptomic responses to viral infections during childhood. MicroRNA expression is altered in nasal secretions during rhinovirus infection, and in particular increases in *hsa-miR-155* may lead to upregulation of genes implicated in the host immune response such as *IL8, DPP7*, and *SOCS1* (46). Rhinovirus-induced wheezing is also associated with reduced *CDHR3* expression in children, which may lead to increased permeability and dysfunction of the airway epithelium (47)—and thus increased susceptibility to infection and immune dysregulation. Another study using cultured primary nasal epithelial cells from children with and without asthma showed that rhinovirus infection affects both DNA methylation and gene expression in children with asthma compared with controls. The authors reported 471 CpG sites with differential methylation that corresponded with genes also showing differential expression, including 16 genes that had CpG sites with >3% difference in methylation and >0.1 RPKM change in gene expression (48). Among those, *BAT3* and *MICB* are genes implicated in natural killer (NK)-cell activation, and *NEU1* has been shown to activate Th2-mediated airway inflammation in mice (48). Therefore, beyond the unbiased search for early biomarkers and previously unknown risk factors, these and other epigenomic studies are helping investigators better understand the pathways that underpin previously known risk factors for asthma and atopic diseases.

To our knowledge only one study has looked at gene expression as a predictor of medication response in asthma. Using gene expression data from 298 subjects aged 12 to 75 years, the authors developed an “eosinophil-related gene signature” panel that correlated with peripheral eosinophil counts and with FEV_1_ improvement after 12 weeks of anti-IL-13 treatment—although the panel did not outperform eosinophil counts or periostin levels (49).

## Strengths, Challenges, and Future Directions

Unlike the genetic code, the methylome and transcriptome are cell-specific and responsive to the environment and change over time and with age—much like the risk of developing asthma or worsening asthma severity. Epigenomic and transcriptomic profiling may thus allow accurate individual-level prediction of asthma risk and response to treatment in subjects with asthma.

Some of the advantages of studying epigenomics and transcriptomics also represent important challenges. Although epigenetic changes can last for many years, some can happen within days. For example, exposure to black carbon air pollution can induce methylation changes in asthma-related genes in children within <5 days (50). The timing and duration of exposure(s) are also important: a recent study in 67 children with asthma and 121 controls showed that the methylation effects of PM_2.5_, NO_2_, and CO on asthma genes may vary according to the level and duration of exposure. Moreover, certain associations differed by age and sex, and the level of the exposures varied depending on the time of the year during which the samples were collected (51). It will also be important to consider cell proportions in samples composed of more than one type of cell, such as white blood cells (WBCs); for example, EWAS of total serum IgE have shown markedly different results depending on whether models were adjusted for WBC types or not (52, 53). Future studies of the epigenomics of asthma will have to consider all these critical aspects, during both study design and data analysis.

To date, most studies of asthma epigenomics and transcriptomics have been cross-sectional, and thus one cannot fully determine whether epigenomic changes are causal or rather a consequence of the disease. Future prospective, longitudinal studies should evaluate whether epigenomic and transcriptomic profiles can serve as predictors or biomarkers. Because asthma is a heterogeneous disease that varies over the life course, “prediction” and “diagnosis” are dynamic concepts. Researchers will thus need to elucidate predictors that identify children at risk for early-onset vs. late-onset asthma, as well as atopic vs. non-atopic asthma; predictors should also ideally identify those who might remit later in life vs. those whose symptoms will persist. Other important areas of study, beyond the scope of the current review, will include the prediction of acute exacerbations and response to medications, with the ultimate aim of providing clinicians with tools to personalize asthma management.

To date studies of atopic asthma have yielded more promising results than those including more “generic” definitions of asthma; further research should elucidate whether this is due to a more rigorous phenotype definition or due to the fact that atopic asthma is more common than non-atopic asthma in children. Finally, rather than studying genomics, epigenomics, or transcriptomics in isolation, it will be critical to integrate multiple “omics” data to improve our understanding of the pathogenesis of asthma (54). Results in the field of epigenomics/transcriptomics have thus far yielded extremely promising results, but much remains to be done.

## Author Contributions

EF wrote the first draft of the manuscript. EF and JC contributed to manuscript revisions. Both authors read and approved the submitted version.

### Conflict of Interest Statement

The authors declare that the research was conducted in the absence of any commercial or financial relationships that could be construed as a potential conflict of interest.
